# Downregulation of the FBXO43 gene inhibits tumor growth in human breast cancer by limiting its interaction with PCNA

**DOI:** 10.1186/s12967-021-03100-0

**Published:** 2021-10-13

**Authors:** Rulan Ma, Kun Zhu, Dawei Yuan, Meijun Gong, Yijun Li, Kang Li, Lei Meng

**Affiliations:** 1 Department of Surgical Oncology, The First Affiliated Hospital of Xi’an Jiaotong University, 277 West Yanta Road, Xi’an, 710061 Shaanxi China; 2 Department of Breast Surgery, The First Affiliated Hospital of Xi’an Jiaotong University, 277 West Yanta Road, Xi’an, 710061 Shaanxi China

**Keywords:** Breast cancer, FBXO43, PCNA, Lentivirus, Tumor growth

## Abstract

**Background:**

The function and regulatory mechanism of FBXO43 in breast cancer (BC) are still unclear. Here, we intended to determine the role and mechanism of FBXO43 in BC.

**Methods:**

FBXO43 expression in BC was evaluated by analysis of The Cancer Genome Atlas (TCGA). RT-qPCR and western blotting were utilized to detect FBXO43 expression in BC cell lines. Lentivirus was applied to downregulate FBXO43 in human BC cells. Proliferation assays were performed to evaluate the proliferative ability of BC cells. The apoptosis and cell cycle analysis of BC cells were analyzed by flow cytometry. Cell migration and invasion were investigated via Transwell assays. The function of FBXO43 in vivo was evaluated by constructing a xenograft mouse model. The proteins that might interact with FBXO43 in BC were identified by mass spectrometry, bioinformatics analysis, and co-immunoprecipitation (Co-IP) assays. Finally, rescue experiments were conducted to validate the recovery effects of the proteins interacting with FBXO43.

**Results:**

FBXO43 was highly expressed in BC and was significantly downregulated after FBXO43 knockdown. The proliferation, migration, and invasion of BC cells were inhibited, and cell apoptosis was induced by FBXO43 knockdown. In addition, an in vivo experiment indicated that FBXO43 knockdown could inhibit the cell growth of BC. The results of the Co-IP assay showed that FBXO43 interacted with PCNA. Further rescue experiments confirmed that overexpression of PCNA significantly reversed the effects of FBXO43 knockdown on BC cells.

**Conclusion:**

Downregulation of FBXO43 inhibits the tumor growth of BC by limiting its interaction with PCNA. FBXO43 might be a new potential oncogene and a therapeutic target for BC.

**Supplementary Information:**

The online version contains supplementary material available at 10.1186/s12967-021-03100-0.

## Introduction

Breast cancer (BC) is currently the most common tumor in the world, and the first leading cause of tumor-related death among women aged 20–59 years [1, 2]. Globally, 2,300,000 new cases were estimated to occur in 2020 [2]. The numbers of new cases and deaths related to BC in the United States in 2021 are estimated to reach 284,200 and 44,130, respectively [1]. Although the 5-year relative survival rate for BC is 90% and the mortality rate has dropped by 40% since 1989, its incidence rates has continuously increased by approximately 0.5% each year [3]. Although systematic treatment strategies, including surgical resection, radiotherapy, chemotherapy, hormone treatment, and immunotherapy, are used in the clinic, the prognosis of BC patients is still not satisfactory. Therefore, it is urgent to further explore the molecular regulatory mechanism of BC to identify more useful biomarkers for prognosis and treatment.

F-box only protein 43 (FBXO43), also named EMI2, is a member of the F-box protein family, which is characterized by an approximately 40-amino acid F-box motif. It was initially founded as a new protein interacting with Plx-1 in a yeast two-hybrid screening and was found to contribute to the arrest of cytostatic factors in vertebrate meiosis [4–6]. FBXO43 was demonstrated to be a key component of cytostatic factor and was recognized as an inhibitor of the anaphase-promoting complex or cyclosome [6]. It was reported that FBXO43 was essential for meiosis in mice and meiosis I progression in spermatocytes [7]. However, only a few studies have focused on the roles of FBXO43 in tumorigenesis and malignant progression.

In 2019, Xu et al. demonstrated via gene co-expression network analysis that 10 genes, including FBXO43, could serve as prognostic and progression biomarkers of hepatocellular carcinoma [8]. Later, histological analysis of the prognostic value of FBXO43 in BC was reported. It was demonstrated that high FBXO43 expression was positively correlated with a worse prognosis and a high risk of metastasis [9]. However, the intrinsic molecular mechanism of FBXO43 in BC is still unknown.

For this study, we constructed lentiviral vectors containing shRNA targeting FBXO43 and transfected human BC cells with lentivirus to elucidate the biological functions of FBXO43 in BC and the underlying mechanism by which FBXO43 regulates tumor growth.

## Materials and methods

### Gene expression analysis

We downloaded the RNA-seq data of patients with BC from The Cancer Genome Atlas (TCGA) database (https://cancergenome.nih.gov/). Then, the expression levels of FBXO43 in BC tumor tissue and adjacent normal tissue were analyzed after filtering and standardizing the data.

### Cell culture

The human BC cell lines MDA-MB-231, MCF7, and T-47D, were obtained from GeneChem Corporation (Shanghai, China). The cells were cultured in DMEM (Corning, 10-013-CVR) with 10% FBS (Ausbian, A11-104) at 37 °C with 5% CO_2_.

### Antibodies

The antibodies used in this study were as follows: anti-FBXO43 (Sigma, HPA024292, 1:500), anti-GAPDH (Santa-Cruz, sc-32233, 1:2,000), anti-Flag (Sigma, F1804, 1:2,000), anti-β-actin (Santa Cruz, sc-69879, 1:2,000), anti-VCP (Abcam, ab109240, 1:10,000), anti-CD44 (Abcam, ab51037, 1:5,000), anti-PCNA (CST, #2586, 1:1,000), anti-ACLY (Abcam, ab40793, 1:1,000), anti-HSPA5 (Abcam, ab108615, 1:1,000), anti-ACTN4 (Abcam, ab108198, 1:1,000), anti-rabbit IgG (Santa-Cruz, sc-2004, 1:2,000), anti-mouse IgG (Santa-Cruz, sc-2005, 1:2,000), anti-rabbit IgG (CST, #7074), and anti-mouse IgG (CST, #7076, 1:10,000).

### Plasmid construction and lentiviral transfection

The shRNA-1 (5′-CAAGTTATCAACTTAGAAA-3′) and shRNA-2 (5′-TTAACACATCCTTTAGAAT-3′) sequences were designed to silence FBXO43, while a scrambled sequence (5′-TTCTCCGAACGTGTCACGT-3′) was used as a negative control. Then, the single-stranded DNA oligos with interfering sequences were synthesized, and double-stranded DNA was synthesized after primer annealing. The restriction enzyme cleavage sites of the double-stranded DNA were directly ligated into lentiviral vectors treated with restriction endonucleases. The ligated products were transformed into competent *E. coli* cells (TIANGEN, #CB104-03), and the positive recombinant vector was identified and verified by quantitative PCR (qPCR). The lentiviral vectors with the correct sequences were extracted and purified by using the EndoFree Midi Plasmid Kit (TIANGEN, #DP118-2) according to the manufacturer’s instructions. Similarly, the lentiviruses were constructed to up-regulate the expression levels of FBXO43, PCNA, VCP and ACLY.

Lentiviral plasmids with interfering sequences were transfected into 293T cells. After 48 h of incubation, the viral supernatant was collected, centrifuged, and purified, and the quality of the lentivirus was detected. Then, fluorescence-labeled lentiviruses were transfected into MDA-MB-231 cell line. When the fluorescence rate exceeded 80% and the cell confluence reached 80%, the cells were collected for further experiments.

### Cell proliferation assay

The transfected human BC cell lines were seeded into 96-well plates (1500 cells/well) and incubated. The number of cells with green fluorescence was detected by Celigo image cytometry (Nexcelom) for 5 days continuously. The cell images were analyzed by Celigo software (Nexcelom).

### MTT assay

Cell viability was evaluated by using MTT solution (Genview, JT343). Briefly, the transfected cells were seeded into 96-well plates (2000 cells/well). 20 ml MTT solution (5 mg/ml) was added to the wells and incubated for four hours. Then, the supernatant was removed, and the formazan was dissolved in 100 μl DMSO (Shi Yi Co., Ltd, 130,701). The absorbance at 490 nm was detected by using an enzyme calibration system (Tecan Infinite, M2009PR).

### Colony formation assay

The transfected cells were seeded in 6-well plates (500 cells/well) and incubated for 8 days when the number of cells in most single clones was greater than 50. The media were changed every three days. Then, the cells were fixed with 4% paraformaldehyde (Sinopharm Chemical Reagent Co., Ltd.) and stained with methyl violet (Sangon Biotech, CB0331). ddH_2_O was used to wash the cells. A microscope (Cai Kang Optical Instrument Co., Ltd, XDS-100) was used to scan and quantify the cell colonies.

### Cell apoptosis

The number of apoptotic cells was detected using a FITC-Annexin V apoptosis detection kit (eBioscience, 88-8007). Briefly, the transfected cells were washed with cold D-Hanks (pH 7.2–7.4) and resuspended in 1 × binding buffer. Then, 10 μl Annexin V-APC was added and incubated for 10–15 min. Finally, a flow cytometer (BD, C6 PLUS) was used to detect the number of apoptotic cells.

### Scratch wound healing assay

Cells were seeded into 6-well plates. When the cell density exceeded 90%, scratches were created across the plates, and the plates were washed with media. Then, the cells were incubated for 24 h. The cell images were photographed at 0 h and 24 h with a fluorescence microscope (Olympus, IX71), and the area of cell migration was analyzed via Celigo image cytometry (Nexcelom).

### Transwell migration and invasion assay

Cell migratory and invasive abilities were evaluated via a Transwell migration kit (Corning, 3433) and Transwell invasion kit (Corning, 354480), respectively, according to the manufacturer's instructions. The infected cells (1 × 10^5^ cells/chamber) were plated into the upper chambers, whereas the lower chambers were supplemented with media with 30% FBS. After 24 h incubation, the medium was discarded, and the non-migrated cells were swabbed. Then, the cells were fixed with 4% paraformaldehyde and stained with crystal violet solution. The count of migratory or invasive cells was counted under a microscope (Olympus, IX71).

### Real-time quantitative PCR (RT-qPCR)

Total RNA from MDA-MB-231 cells was obtained by using TRIzol Reagent (Shanghai Pufei Biotech Co., Ltd, #3101-100) and was reverse transcribed into cDNA by using M-MLV Reverse Transcriptase (Promega, M1705) according to the manufacturer’s protocols. qPCR was performed by using SYBR Master Mixture (TAKARA, DRR041B) on a real-time PCR instrument (Roche, LightCycler 480 II). The following primer sequences were used in this study: FBXO43: 5′-CTCCGATAAGTAATCTTGTGGC-3′ (upstream) and 5′-CTTGTCTTTCTTATGGTGTCCC-3′ (downstream); PCNA: 5′-TGAAGCACCAAACCAGGAG-3′ (upstream) and 5′-GAAGGCATCTTTACTACACAGC-3′ (downstream); VCP: 5′-TCTGATGATACTTGTTCTGATGAG-3′ (upstream) and 5′-ATGGCTGGATGCTGATGAC-3′ (downstream); ACLY: 5′-GCGATACCATCTGTGATCTAG-3′ (upstream) and 5′-TTGTGACTTCGTGCTCCTT-3′ (downstream); GAPDH: 5′-TGACTTCAACAGCGACACCCA-3′ (upstream) and 5′-CACCCTGTTGCTGTAGCCAAA-3′ (downstream). The relative gene expression levels were calculated according to the 2^−ΔΔCt^ method.

### Western blot analysis

The cells were washed with PBS and lysed with cold 2 × lysis buffer for 10 min. The BCA Protein Assay Kit (Beyotime Biotechnology, P0010S) was used to detect the abundance of proteins in the supernatant of cell lysate. Then, the proteins were separated on a 10% SDS–polyacrylamide gel (Tanon Science & Technology Co., Ltd., VE-186) and transferred to a polyvinylidene difluoride membrane (Millipore, IPVH00010). The membranes were blocked with PBST with 5% nonfat milk and incubated with antibodies. An ECL-PLUS/Kit (Thermo, M3121/1859022) was used to detect the protein bands. Quantitative analysis was conducted by ImageJ (https://imagej.nih.gov/ij/).

### Detection of interacting proteins by co-immunoprecipitation (Co-IP) assay and mass spectrometry

Lentiviruses containing 3 × Flag-FBXO43 were constructed to stably overexpress 3 × Flag-FBXO43 and were used to transfected MDA-MB-231 cells. The expression level of 3 × Flag-FBXO43 was detected using RT-qPCR and western blot analysis.

The proteins from the transfected MDA-MB-231 cells were extracted with IP buffer (Beyotime Biotechnology, P0013). The cell lysate, PBS, and Flag beads were incubated for twenty-four hours. The mixture was centrifuged and washed with TBS, and the supernatant was discarded. Then, 3 × Flag peptide (Sigma, F4799) and TBS were added and incubated for 40 min at 4 °C. After the mixture was centrifuged and concentrated, loading buffer was added. The reactive product was separated on a 10% SDS–polyacrylamide gel (Tanon Science & Technology Co., Ltd., VE-186) and stained with Coomassie Brilliant Blue. Finally, western blotting was performed.

The gel strips pulled down with 3 × Flag were cut off, and the proteins in each gel strips were digested and cut into peptides with trypsin (Promega, V5117). Each peptide sample was detected by shotgun liquid chromatograph-mass spectrometer. Proteome Discoverer 2.1 (Thermo) and MASCOT 2.5 (Matrix Science) software were used to obtained the results of protein identification by checking the database. Finally, the bioinformatics analysis of the identified protein list was performed.

### Animal study

The animal study was performed according to the guidelines of the laboratory Animal Care Committee of Xi’an Jiaotong University. Four-week-old BALB/c nude mice were obtained from Charles River Laboratories and subcutaneously injected with 1 × 10^7^ MDA-MB-231 cells transfected with lentiviral vector containing shRNA-1. When the length of the tumor exceeded 5 mm, the tumor volume (π/6 × length × width^2^) and mouse weight were detected twice every week. The mice were sacrificed three weeks later. In vivo imaging was performed to detect the total fluorescence intensity before sacrifice.

### Cell cycle analysis

The transfected MDA-MB-231 cells were centrifuged for 5 min (1300 rmp/min) and the supernatant was discarded. The cells were washed with precooled DPBS (pH = 7.2–7.4) and centrifuged for 5 min (1300 rmp/min). The cells were stained with 40 × PI solution (2 mg/ml) (Sigma, P4170), 100 × RNase A (10 mg/ml) (Thermo Fisher Scientific, EN0531), 1 × DPBS and Triton X-100 (Sigma, SLBT4524) (the ratio of stain solution is 25:10:1000:40). The stained cells were detected using flow cytometry and the cell pass rate was 300 ~ 800 cell/s. Cell cycle was analyzed by ModFit software (Millipore, Guava easyCyte HT).

### Statistical analysis

Data analysis was conducted via GraphPad Prism 8.3.0 (GraphPad Software, Inc.), IBM SPSS 22.0 (IBM, Corp.) and R version 3.0.3. Data were presented as the mean (M) ± standard error of the mean (SEM). Statistical differences were calculated by t-test or one-way ANOVA. Differences with a *P*-value < 0.05 were considered statistically significant.

## Results

### FBXO43 was overexpressed in human BC and downregulated by RNA interference

**Fig. 1:**
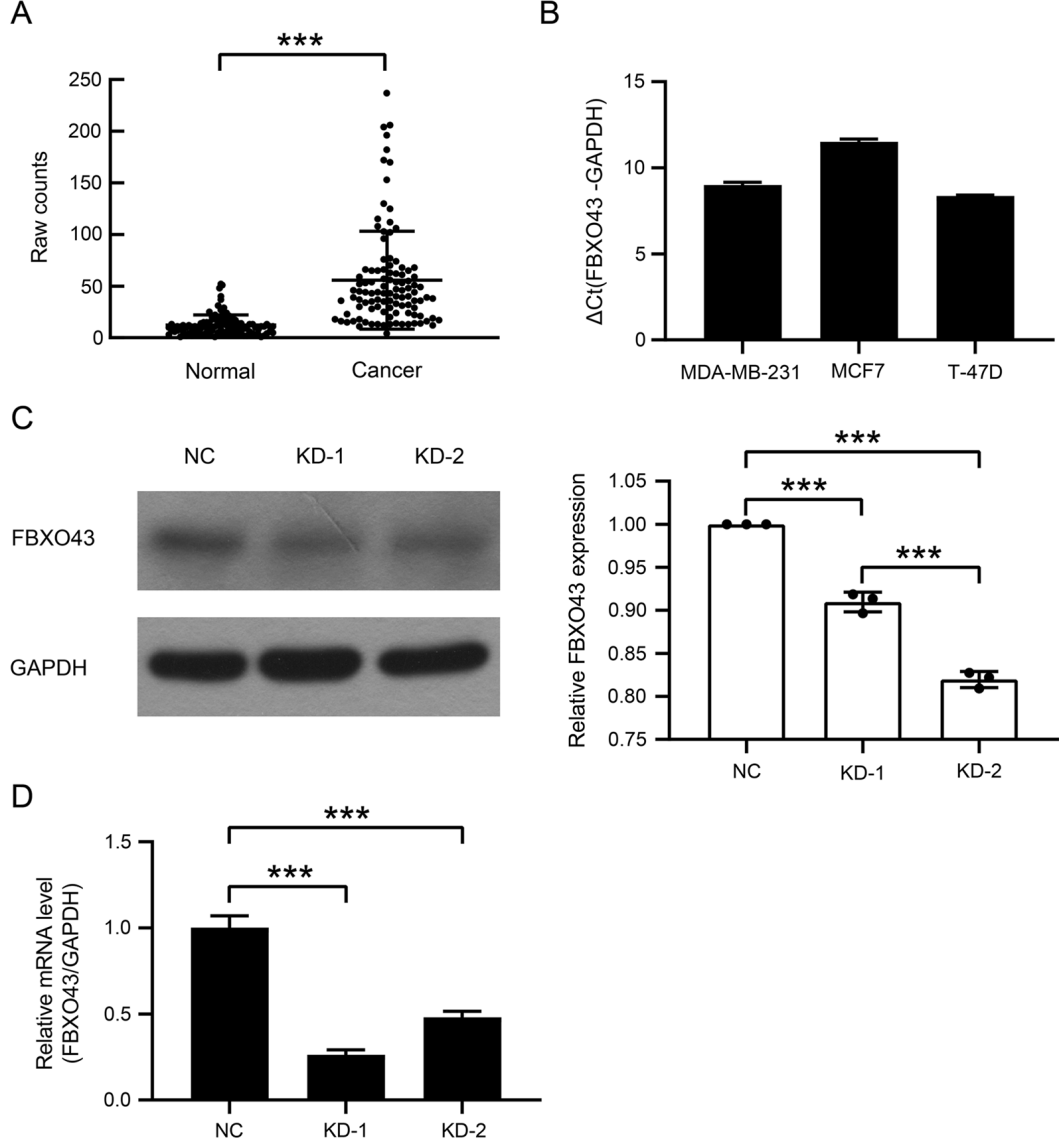
FBXO43 was highly expressed in human breast cancer and was inhibited by lentiviral infection with shRNA against FBXO43. **A** FBXO43 expression levels in human breast invasive carcinoma and adjacent normal tissues according TCGA analysis. **B** FBXO43 expression levels in human breast cancer cell lines according to RT-qPCR. **C** FBXO43 expression levels in MDA-MB-231 cells infected with lentivirus containing shRNA against FBXO43 and negative control lentivirus according to western blot analysis and the quantitative analysis. **D** mRNA expression levels of FBXO43 in infected MDA-MB-231 cells according to RT-qPCR. All experiments were performed at least three times. *NC* negative control, *KD* FBXO43 knockdown. *P < 0.05, **P < 0.01, ***P < 0.001

A previous study showed that FBXO43 expression was associated with a lower survival rate and metastasis in BC patients [9]. Therefore, to further validate the function of FBXO43 in BC, we estimated the expression level of FBXO43 by TCGA analysis and found that the expression level of FBXO43 was significantly higher in tumor tissues than in adjacent normal tissues in BC patients (Fig. 1A). RT-qPCR assays also showed that FBXO43 was overexpressed in human BC cell lines, including MDA-MB-231, MCF7, and T-47D cells (Fig. 1B).

We next transfected MDA-MB-231 cells with lentiviruses containing shRNA to silence FBXO43 or lentiviruses containing a scrambled shRNA. Western blot assay and RT-qPCR analysis showed that FBXO43-specific shRNA significantly inhibited FBXO43 expression in MDA-MB-231 cells (Fig. 1C, D).

### Downregulation of FBXO43 inhibited the proliferation of BC cells

**Fig. 2:**
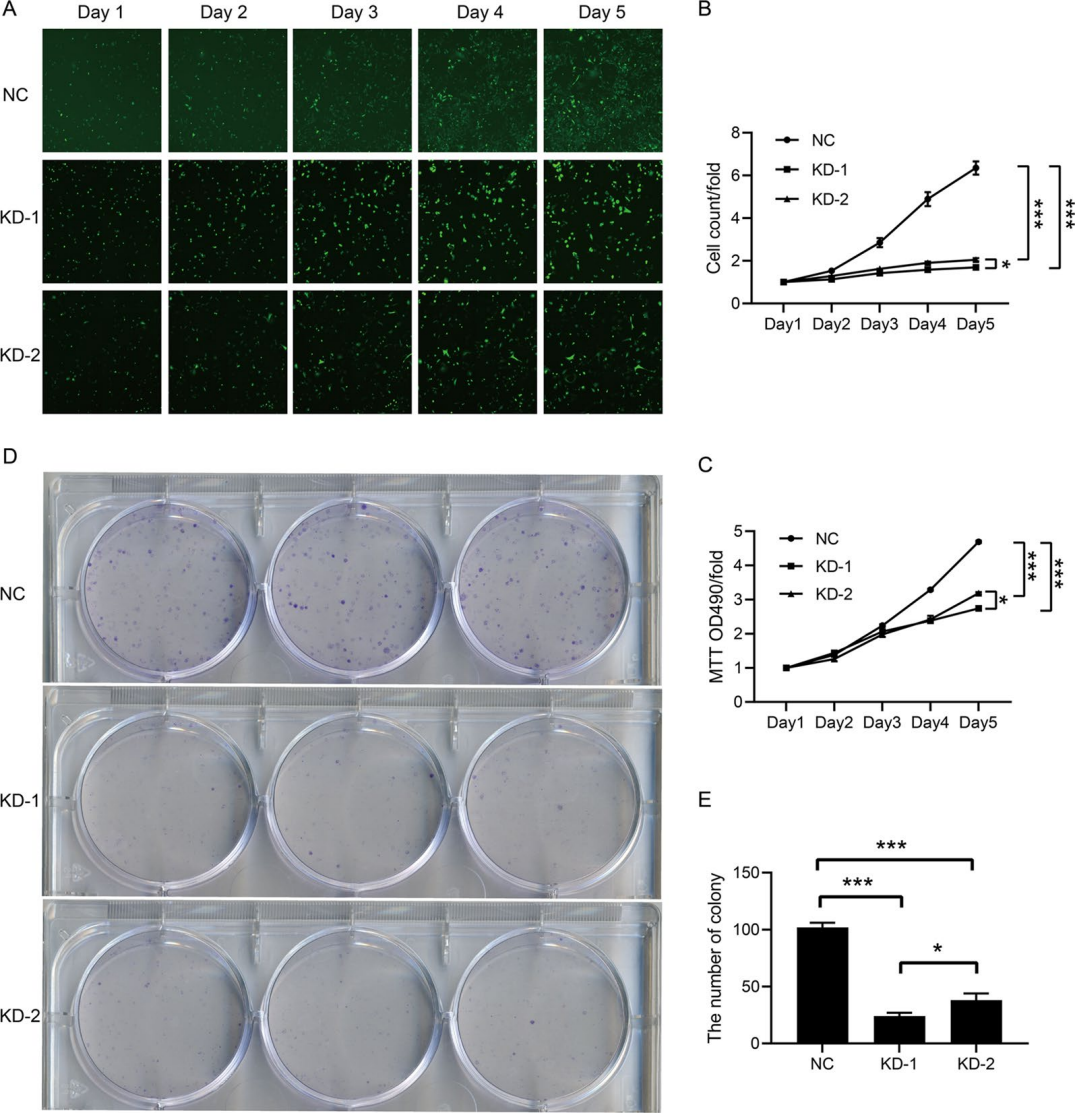
FBXO43 knockdown inhibited the proliferation of MDA-MB-231 cells. **A**, **B** Celigo image cytometry. **C** MTT assay. **D**, **E** Colony formation assay. All experiments were performed at least three times. All cell images were obtained with a microscope at 100 × magnification. *NC* negative control, *KD* FBXO43 knockdown. *P < 0.05, **P < 0.01, ***P < 0.001

To clarify the effect of FBXO43 interference on the proliferation of BC cells, we performed several proliferation assays. We found that, compared with the negative control, FBXO43 knockdown significantly restrained the growth of MDA-MB-231 cells (Fig. 2A, B). The MTT assay also showed the inhibitory effect of FBXO43 interference on the viability of MDA-MB-231 cells (Fig. 2C). In addition, a colony-formation assay revealed that FBXO43 knockdown was positively correlated with a decrease in the colony-forming ability of MDA-MB-231cells (Fig. 2D, E). All the findings indicated that FBXO43 interference suppressed the growth and decreased the viability of MDA-MB-231 cells.

### Downregulation of FBXO43 induced the apoptosis of BC cells

**Fig. 3:**
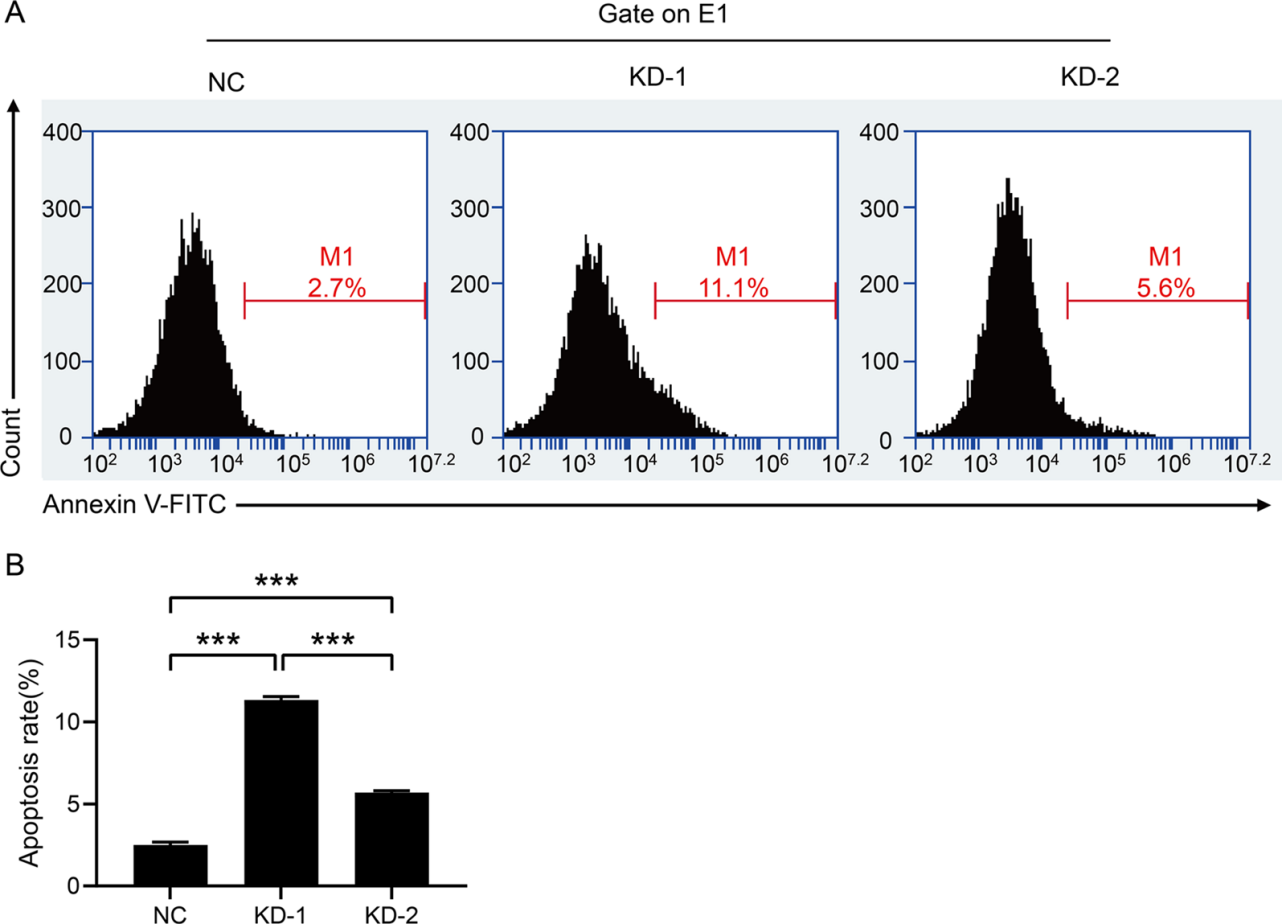
Flow cytometry analysis showed that FBXO43 knockdown promoted the apoptosis of MDA-MB-231cells. **A** Representative images of flow cytometry assay. **B** Quantitative analysis of the result. All experiments were performed at least three times. *NC* negative control, *KD* FBXO43 knockdown. *P < 0.05, **P < 0.01, ***P < 0.001

Given that FBXO43 knockdown suppressed the proliferation of MDA-MB-231 cells, we investigated whether FBXO43 knockdown influenced cell apoptosis. We performed a flow cytometry assay and found that the number of apoptotic cells was increased after FBXO43 knockdown (Fig. 3A, B).

### Downregulation of FBXO43 inhibited the migratory and invasive ability of BC cells

**Fig. 4:**
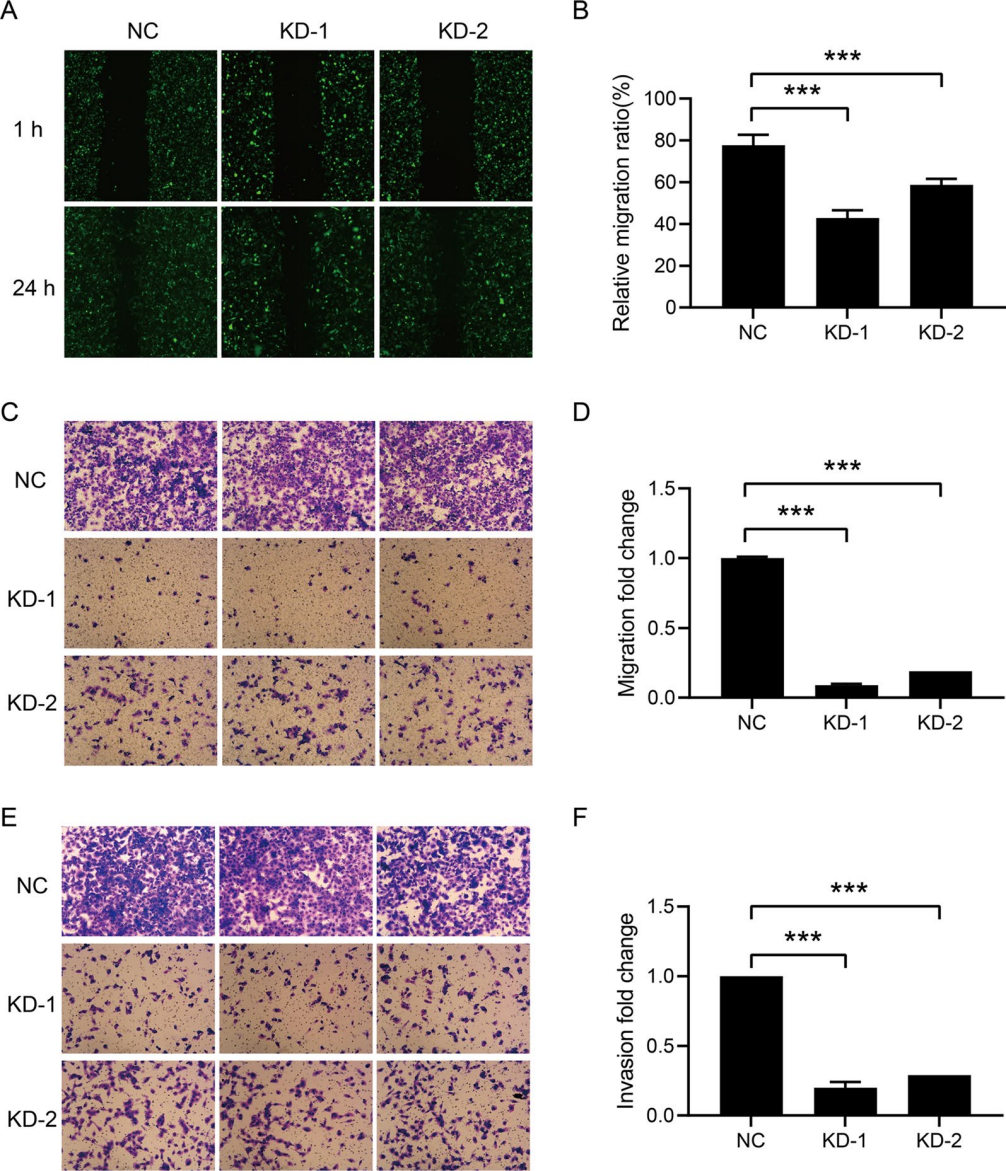
FBXO43 knockdown inhibited the migration and invasion of MDA-MB-231 cells. **A**, **B** Scratch wound healing assay. **C**, **D** Transwell migration assay (without ECM). **E**, **F** Transwell invasion assay (with ECM). All experiments were performed at least three times. All cell images were obtained under a microscope at 100 × magnification. *ECM* extracellular matrix, *NC* negative control, *KD* FBXO43 knockdown. *P < 0.05, **P < 0.01, ***P < 0.001

To investigate the effects of FBXO43 on the migration and invasion of BC cells, we performed scratch wound healing and Transwell assays. The wound healing assay results showed that the migratory area was decreased after FBXO43 interference (Fig. 4A, B). In addition, the Transwell migration assay showed that the migratory ability of MDA-MB-231 cells was significantly inhibited by FBXO43 knockdown (Fig. 4C, D). Transwell invasion assays were conducted to detect the role of FBXO43 interference on the invasion of MDA-MB-231 cells. The invasive ability of MDA-MB-231 cells transfected with lentivirus containing FBXO43-specific shRNA was significantly suppressed (Fig. 4E, F). Taken together, these findings indicated that FBXO43 knockdown suppressed the invasion and migration of MDA-MB-231 cells.

### Downregulation of FBXO43 inhibited the tumor growth of BC in vivo

**Fig. 5:**
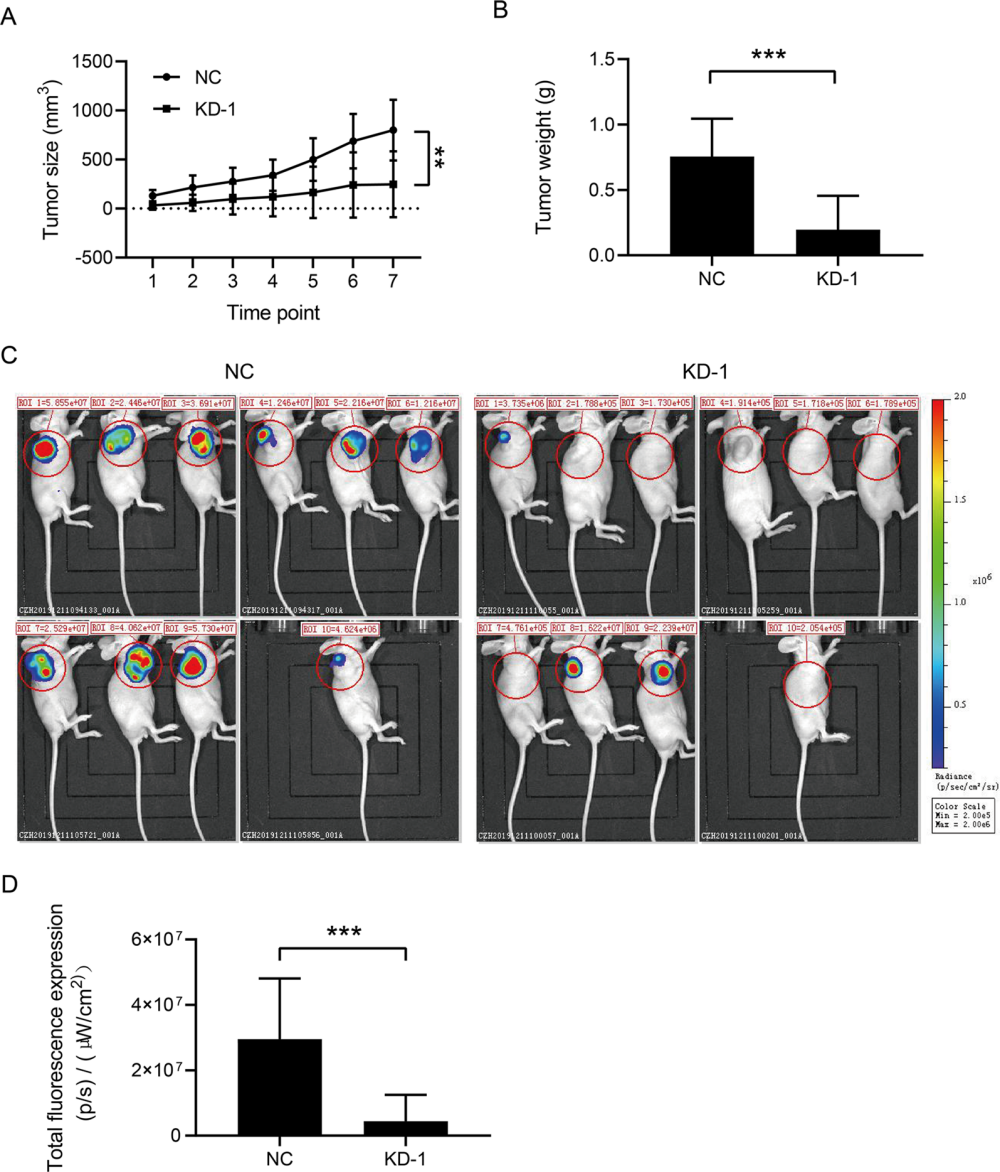
FBXO43 knockdown inhibited the growth of breast cancer tumors in vivo. **A**, **B** Mouse xenograft assays showed the tumor volume and weight in the negative control group and the knockdown group. **C**, **D** Detection of tumor growth by bioluminescence imaging of luciferase. *NC* negative control, *KD* FBXO43 knockdown. *P < 0.05, **P < 0.01, ***P < 0.001

Given that FBXO43 interference inhibited the proliferation, migration, and invasion of BC cells in vitro, we next created a nude mouse xenograft model with subcutaneous injection of transfected MDA-MB-231 cells to clarify the function of FBXO43 in vivo. We found that the tumor volume and weight in the FBXO43 knockdown group were less than those in the negative group (Fig. 5A, B). In addition, in vivo imaging showed that, compared with that in the negative control group, the total fluorescence intensity in the FBXO43 knockdown group was lower (Fig. 5C, D). Collectively, FBXO43 knockdown obviously restrained the growth of BC in vivo.

### Downregulation of FBXO43 inhibited the proliferation and migration of BC cells by limiting its interaction with PCNA

**Fig. 6:**
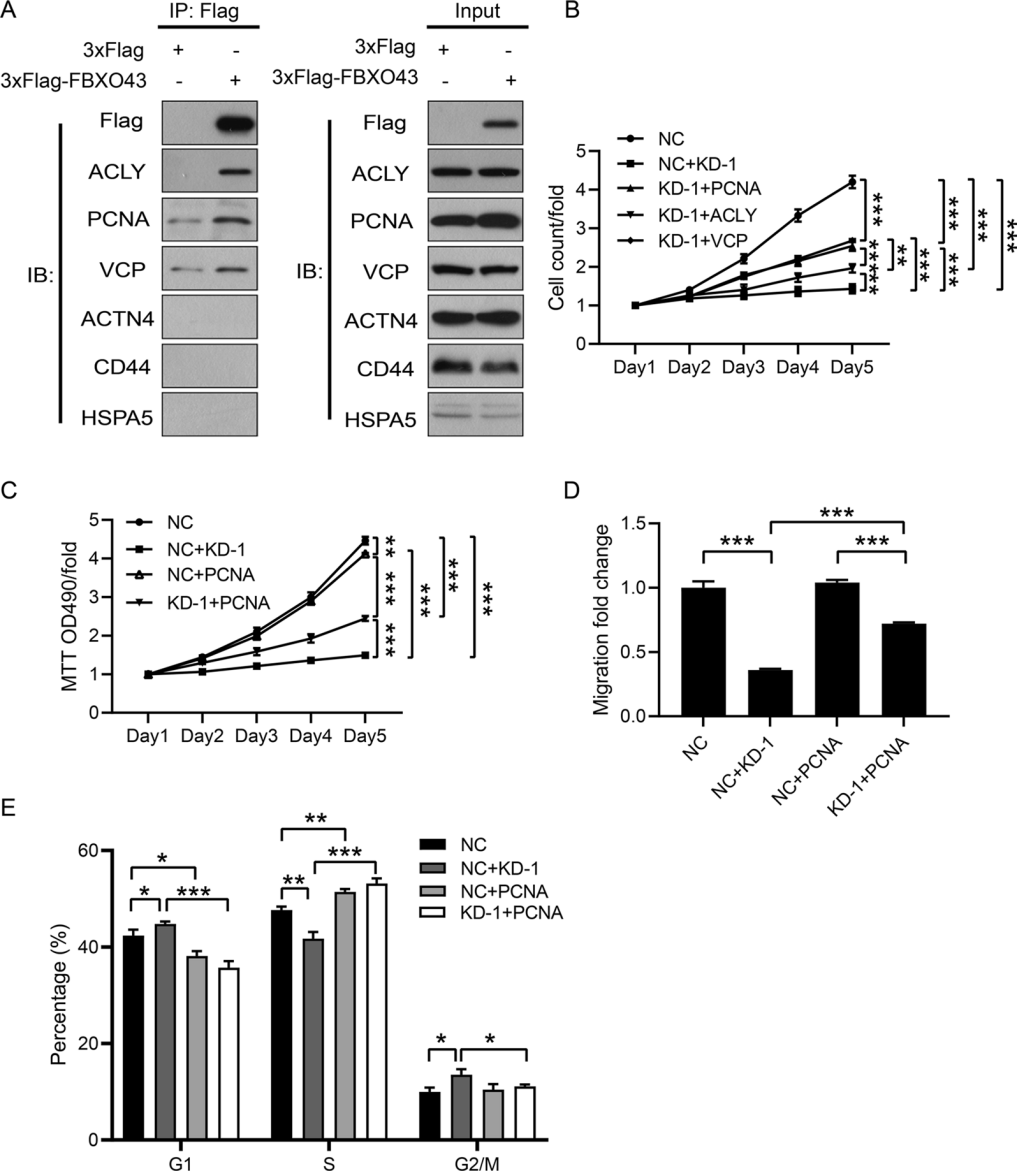
FBXO43 knockdown inhibited the proliferation and invasion of breast cancer cells by suppressing PCNA. **A** Co-IP analysis showed that ACLY, PCNA and VCP interacted with FBXO43. **B** HCS assay. **C** MTT assay. **D** Transwell migration assay (without ECM). **E** Cell cycle analysis. All experiments were performed at least three times. ECM, extracellular matrix. NC: negative control. KD-1 + NC: FBXO43 knockdown + negative control. KD-1 + ACLY: FBXO43 knockdown + ACLY overexpression. NC + PCNA: negative control + PCNA overexpression. KD-1 + PCNA: FBXO43 knockdown + PCNA overexpression. KD-1 + VCP: FBXO43 knockdown + VCP overexpression. *P < 0.05, **P < 0.01, ***P < 0.001

To explore the intrinsic molecular mechanism by which FBXO43 regulated the growth of BC, we constructed lentiviruses to stably overexpress 3 × Flag-FBXO43 (Additional files 1, 2: Figure S1A, Figure S2A) and conducted a Flag-tagged pull-down assay to identify the proteins interacting with FBXO43 (Additional file 2: Figure S2B). We identified 288 that might interact with FBXO43 (data not shown) via mass spectrometry. TGCA data analysis showed 66 proteins among the above 288 proteins was related to BC. Next, bioinformatics analysis was performed and the interactions of FBXO43 and 66 identified proteins that was related to the proliferation and metastasis of BC were estimated (Additional file 2: Figure S2C). Six proteins (ACLY, PCNA, CVP, ACTN4, CD44, HSPPA5) that was related to proliferation and metastasis of BC were selected for the Co-IP assay to verify the findings of mass spectrometry analysis. ACLY, PCNA, and VCP were found to interact with FBXO43 (Fig. 6A).

To investigate whether the overexpression of the ACLY, PCNA or VCP gene had a functional recovery effect on FBXO43 knockdown cells, we constructed lentiviruses for overexpressing ACLY, PCNA and VCP, co-transfected MDA-MB-231 cells with these lentiviruses and lentivirus containing FBXO43-specific shRNA, and performed the following rescue experiments. RT-qPCR showed that the expression levels of ACLY, PCNA and VCP were elevated after overexpression (Additional file 1: Figure S1B–D). High-content screening (HCS) proliferation assay indicated that ACLY, PCNA, or VCP overexpression in BC cells transfected with lentivirus containing FBXO43-specific shRNA reversed the suppressive effect of FBXO43 knockdown on the proliferation (Fig. 6B). Further MTT assays showed that overexpression of PCNA significantly abated the inhibitory effect of FBXO43 interference on BC cell viability (Fig. 6C). In addition, invasion assays validated that reintroduction of PCNA could obviously reverse the migration-inhibiting effects of FBXO43 knockdown (Fig. 6D). Given that FBXO43 and PCNA are involved in the process of cell division and proliferation [6, 7, 10], we performed cell cycle analysis to confirm the effect of FBXO43 and PCNA on the cell division of MDA-MB-231 cells. The result showed that knockdown of FBXO43 inhibit the cell division, whereas overexpression of PCNA significantly reverse the inhibition of FBXO43 knockdown on MDA-MB-231 cell division (Fig. 6E). All the findings demonstrated that downregulation of FBXO43 inhibited the tumor growth of BC by limiting its interaction with PCNA.

## Discussion

Although the five-year survival rate of BC in high-income countries has reached 85–90%, its prognosis in many low/middle-income countries is still poor [11]. The incidence of BC has been continuously increasing in many countries worldwide [3, 12–14]. Therefore, it is necessary to deeply explore the pathogenesis of BC and identify more useful biomarkers for treatment and prognostic prediction.

As an inhibitor of the anaphase-promoting complex or cyclosome, FBXO43 has been demonstrated to be essential for the meiotic division of oocytes and spermatocytes [7]. As for the roles of FBXO43 in tumors, a previous study reported that elevated expression of FBXO43 was correlated with tumor size, lymph node metastasis, and poor survival in BC patients [9]. However, this study did not reveal the mechanism by which FBXO43 regulates the development of BC. In our study, TCGA data analysis showed that FBXO43 was highly expressed in human BC tissues, which was in accordance with the results of a previous study [9]. The RT-qPCR results also showed elevated FBXO43 expression in human BC cell lines. Subsequently, we observed that the downregulation of tumor growth and the promotion of tumor apoptosis. Additionally, an in vivo experiment demonstrated that the tumor size and weight were significantly suppressed by FBXO43 knockdown. These results indicated that FBXO43 might be a potential oncogene and that FBXO43 interference is likely to help control the occurrence and development of BC.

Next, to further elucidate the mechanism by which FBXO43 affect cell functions in BC, we conducted a Co-IP assay and found that FBXO43 could regulate tumor growth by interacting with PCNA. Rescue experiments further demonstrated that overexpression of PCNA significantly abated the inhibitory effect of FBXO43 knockdown on the cell growth and migration of human BC cells. The above results confirmed the crucial roles of FBXO43 and PCNA in modulating the progression of BC.

Cell proliferation ability is an important prognostic biomarker in cancer diagnosis. PCNA is considered a marker of proliferation and DNA replication. It was found for the first time in the nuclei of mitotic cells of patients with systemic lupus erythematosus [15, 16]. At the same time, another research group found a protein that was produced during the cell cycle and named this protein cyclin [10]. Subsequent studies demonstrated that PCNA and cyclin were the same proteins. PCNA is expressed in the cell division process of normal and malignant cells, and its prognostic and predictive value in malignancies have been evaluated. PCNA was first reported to be associated with human BC prognosis in 1993 [17–22]. Elevated tyrosine 211 (Y211)-phosphorylated PCNA was shown to be correlated with a poor prognosis in patients with BC [23]. Later, researchers found that the combination of PCNA with c-Abl was facilitated by Y211 phosphorylation of PCNA [24]. c-Abl was found to be required for PCNA chromatin association and nuclear foci formation in BC cells with DNA damage. Inhibition of Y211 phosphorylation of PCNA via a cell-penetrating peptide suppressed the interaction of PCNA and Abl [24]. In 2013, Yu et al. [25] also verified that cell growth was suppressed and apoptosis was induced in triple-negative BC cells after inhibition of Y211 phosphorylation of PCNA. In addition, Li et al. [26] found that trifluridine inhibited the growth and induced the apoptosis of triple-negative BC cells by downregulating PCNA. All the previous findings indicate the essential role of PCNA in the tumor regulation of BC, which perhaps explains why overexpression of PCNA had a significant recovery effect on tumor growth inhibition induced by FBXO43 interference.

It has been reported that many PCNA-binding proteins, especially cancer-associated proteins, are intrinsically disordered proteins or have intrinsically disordered regions [27, 28]. These proteins interact with PCNA via a small region containing a conserved motif, named the PCNA-interacting protein-sequence (PIP-box), to regulate PCNA stability, thereby modulating DNA replication and DNA repair [29, 30]. In our study, we observed that FBXO43 was involved in tumor growth and interacted with PCNA in BC. However, whether FBXO43 is an intrinsically disordered protein or whether FBXO43 binds to PCNA via the PIP-box was not clarified in this study. Therefore, more efforts are needed to investigate the binding site by which FBXO43 interacts with PCNA.

In conclusion, our findings well explained why high expression of FBXO43 is related to a worse prognosis in BC patients [9]. Downregulation of FBXO43 inhibits the BC tumor growth by limiting its interaction with PCNA. Therefore, FBXO43 might be a potential oncogene and a target for the treatment of BC.

## Supplementary Information

**Additional file 1: Figure S1**. The expression levels of FBXO43, ACLY, PCNA and VCP in MDA-MB-231 cells transfected with lentiviruses for overexpressing FBXO43, ACLY, PCNA and VCP via RT-qPCR. All experiments were performed at least three times. NC: negative control. FBXO43 (OE): FBXO43 overexpression. ACLY (OE): ACLY overexpression. PCNA (OE): PCNA overexpression. VCP (OE): VCP overexpression. * *P* < 0.05, ** *P* < 0.01, *** *P* < 0.001.

**Additional file 2: Figure S2**. Results of mass spectrometry and bioinformatics analysis. (A) Overexpression of FBXO43 was detected by western blotting. The experiments were performed at least three times. (B) Flag-tagged pull-down assay showed 3 × Flag-FBXO43 was purified. (C) The interaction network of FBXO43 and 66 identified proteins that might interact with FBXO43 by bioinformatics analysis. NC: negative control. FBXO43 (OE): FBXO43 overexpression.

## Data Availability

Data are contained within in this paper.

## Abbreviations

BC: Breast cancer
FBXO43: F-box only protein 43
TCGA: The Cancer Genome Atlas
Co-IP: Co-immunoprecipitation
RT-qPCR: Real-time quantitative PCR
M: Mean
SEM: Standard error of the mean
HCS: High-content screening
PIP-box: PCNA-interacting protein-sequence
Y211: Tyrosine 211
