# Supplementary figures and images for: Downregulation of the FBXO43 gene inhibits tumor growth in human breast cancer by limiting its interaction with PCNA
Source: J Transl Med. 2021 Oct 13;19:425. doi: 10.1186/s12967-021-03100-0 (PMC8513237; doi:10.1186/s12967-021-03100-0)

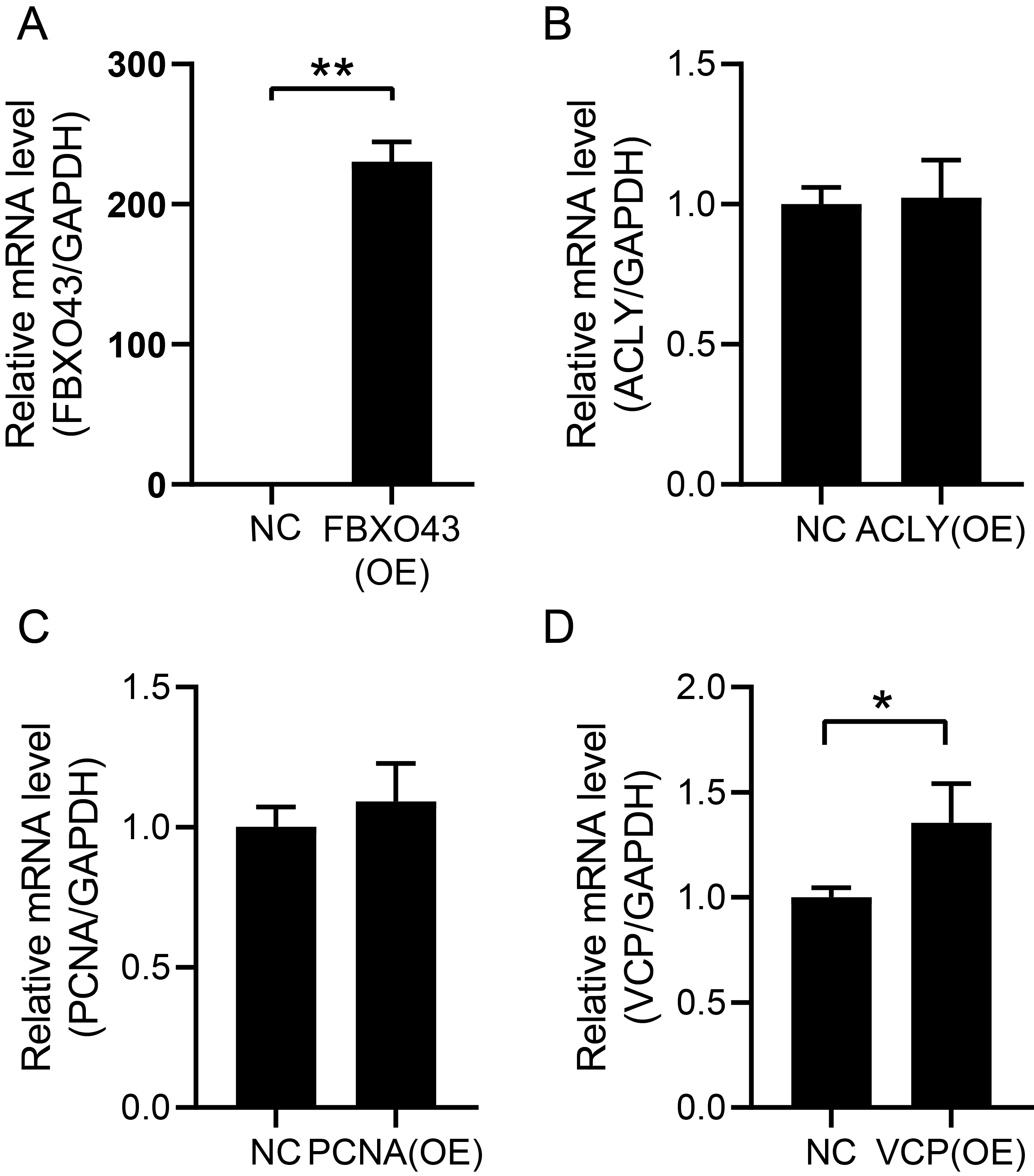

Supplement: Supplementary file 1 — Additional file 1: Figure S1. The expression levels of FBXO43, ACLY, PCNA and VCP in MDA-MB-231 cells transfected with lentiviruses for overexpressing FBXO43, ACLY, PCNA and VCP via RT-qPCR. All experiments were performed at least three times. NC: negative control. FBXO43 (OE): FBXO43 overexpression. ACLY (OE): ACLY overexpression. PCNA (OE): PCNA overexpression. VCP (OE): VCP overexpression. * P < 0.05, ** P < 0.01, *** P < 0.001. [file 12967_2021_3100_MOESM1_ESM.png]

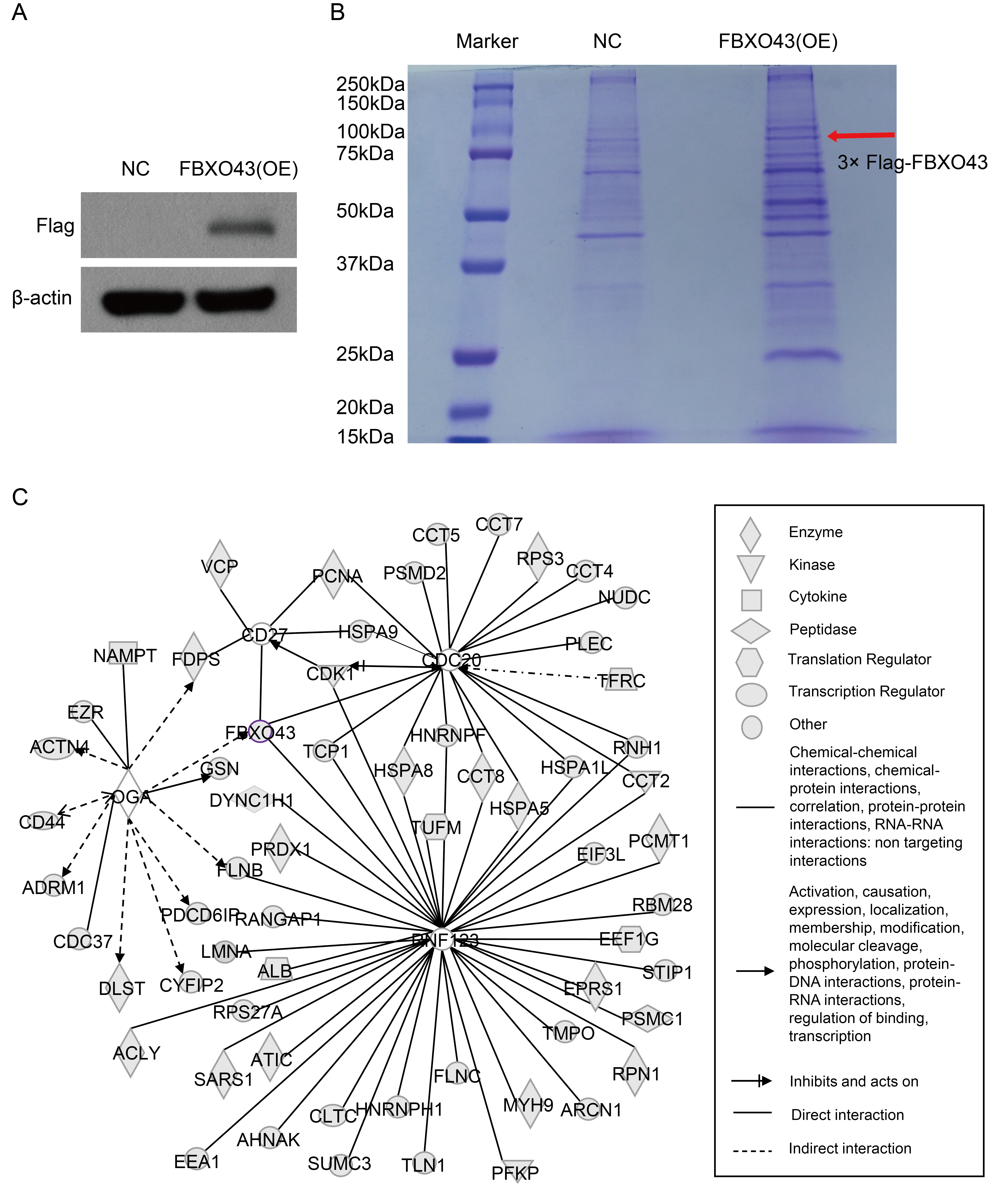

Supplement: Supplementary file 2 — Additional file 2: Figure S2. Results of mass spectrometry and bioinformatics analysis. (A) Overexpression of FBXO43 was detected by western blotting. The experiments were performed at least three times. (B) Flag-tagged pull-down assay showed 3 × Flag-FBXO43 was purified. (C) The interaction network of FBXO43 and 66 identified proteins that might interact with FBXO43 by bioinformatics analysis. NC: negative control. FBXO43 (OE): FBXO43 overexpression. [file 12967_2021_3100_MOESM2_ESM.png]
